# Development and internal validation of a nomogram and machine-learning models for postoperative recurrence in adult patients with chronic rhinosinusitis with nasal polyps

**DOI:** 10.3389/fmed.2026.1884376

**Published:** 2026-08-20

**Authors:** Lin Song, Xiaoli Zhang, Mingliang Feng, Xia Deng, Xiaoli He, Hong Jiang, Lihua Yu, Hui Shi, Jingting Pi

**Affiliations:** 1 Department of Clinical Laboratory, The Affiliated Yongchuan Hospital of Chongqing Medical University, Chongqing, China; 2 General Practice School of Chongqing Medical University, Chongqing, China; 3 Department of Otolaryngology Head and Neck Surgery, The Affiliated Yongchuan Hospital of Chongqing Medical University, Chongqing, China; 4 Department of Neurosurgery, The Affiliated Yongchuan Hospital of Chongqing Medical University, Chongqing, China

**Keywords:** chronic rhinosinusitis with nasal polyps, machine learning, nomogram, postoperative recurrence, predictive model, systemic coagulation–inflammation index

## Abstract

**Background:**

Practical preoperative tools for estimating postoperative recurrence in chronic rhinosinusitis with nasal polyps (CRSwNP) remain limited. We developed and internally validated a conventional nomogram and multiple machine-learning models and evaluated the systemic coagulation–inflammation index (SCI) as a deterministic nonlinear composite of routine laboratory measurements.

**Methods:**

The analysis included 245 adults. To preserve the integrity of the originally locked internal validation, the original outcome-stratified assignments were retained after age exclusions, leaving 170 patients in the training set and 75 in the same-center internal held-out set. Centered VIFs were calculated with an intercept, component-versus-composite SCI models were compared, and ten algorithms were tuned by five-fold stratified cross-validation in the training set. A Firth logistic sensitivity analysis forced hypertension and diabetes into the primary model.

**Results:**

Recurrence occurred in 95/245 patients (38.8%). SCI was the only initial candidate with VIF > 10 (10.259); after its structural exclusion, all remaining VIFs were ≤1.515. In the five-variable multivariable screening model, WBC was inversely associated with recurrence (OR 0.437, 95% CI 0.307–0.623), whereas PLT (OR 1.024, 95% CI 1.013–1.035) and FIB (OR 4.480, 95% CI 2.346–8.557) were positively associated (all *p* < 0.001). The final three-variable nomogram had a held-out AUC of 0.872 and Brier score of 0.149. Hypertension and diabetes data were available for 243/245 adults; forcing both comorbidities into a Firth sensitivity model did not materially change the three primary associations or improve held-out AUC (0.862 versus 0.872; DeLong *p* = 0.565). SCI did not show stable incremental held-out value beyond its components.

**Conclusion:**

A nomogram based on WBC, PLT, and FIB showed promising same-center internal performance in adults. SCI should be interpreted as a deterministic nonlinear composite rather than unique biological information. External validation in cohorts with standardized tissue histopathology and comprehensive comorbidity data is required before clinical use.

## 1 Introduction

Chronic rhinosinusitis with nasal polyps (CRSwNP) is a prevalent chronic inflammatory condition, affecting approximately 1% to 4% of the global population (1). Its main clinical manifestations include nasal congestion, diminished or absent olfactory function, headache, and facial pressure. It can lead to potentially severe complications involving the ocular or central nervous systems, which significantly impairs patients’ quality of life and imposes substantial socioeconomic burdens on healthcare systems and communities (2). Currently, medical interventions such as intranasal glucocorticoids, antibiotics, and nasal irrigation can alleviate symptoms to a certain extent, but for those with insufficient response, Endoscopic Sinus Surgery (ESS) remains the first-line surgical treatment option (3). However, although ESS can significantly improve clinical symptoms, postoperative recurrence rates remain high: studies show recurrence rates of 20–60% from 18 months to 4 years after surgery (4), and up to 79% after 12 years of follow-up (5); in patients with asthma, the recurrence rate can approach 100% (6). These data highlight the urgent need to identify preoperative biomarkers that can accurately predict postoperative recurrence risk, which would help tailor individualized treatment plans and improve long-term prognosis.

The pathogenesis of CRSwNP is not yet fully understood. Existing studies indicate involvement of immune dysregulation, local inflammatory responses, and tissue remodeling among multiple mechanisms (7, 8), with persistent inflammatory cell infiltration and activation of proinflammatory mediators considered key factors in polyp formation (9). Moreover, recent research has found that increased activation of the coagulation cascade and reduced fibrinolysis lead to excessive fibrin deposition, thereby promoting polyp formation (10). The increased fibrin deposition partly results from downregulation of tissue-type plasminogen activator (tPA) and decreased fibrinolytic activity, suggesting that coagulation and fibrinolytic systems also play important roles in the development and progression of CRSwNP (11). These findings provide new perspectives on the complex pathological mechanisms of CRSwNP.

Composite inflammatory indices, including the systemic inflammation response index (SIRI) and systemic immune–inflammation index (SII), have been studied across inflammatory conditions (12–14) and in pediatric or adult CRS cohorts (15, 16). The systemic coagulation–inflammation index (SCI), calculated deterministically as fibrinogen × platelet count / leukocyte count, has also been evaluated in cardiovascular and respiratory diseases (17, 18). Because SCI contains no measurement beyond its three source variables, any apparent contribution should be interpreted as the behavior of a specific nonlinear composite rather than as unique biological information.

We integrated preoperative clinical, laboratory, imaging, and endoscopic variables to develop a conventional logistic nomogram and ten machine-learning models. In addition to evaluating internal discrimination, calibration, and clinical utility, we examined SCI using component, composite, and expanded sensitivity models to determine whether its predictive behavior was consistent beyond that of WBC, PLT, and FIB.

## 2 Materials and methods

### 2.1 Study population

This retrospective study was conducted at The Affiliated Yongchuan Hospital of Chongqing Medical University and was approved by the local Ethics Committee (Approval No. 2025EC0220). Written informed consent was obtained from the participants or their authorized representatives. The source worksheet contained 301 physical rows, 3 of which were completely blank and were not counted as patient records. Of the 298 patient records assessed, 26 patients aged ≤18 years were excluded. Among the remaining 272 age-eligible adults, 27 had no numeric C-reactive protein value available for complete-case analysis. The final analytical cohort therefore comprised 245 adults, including 95 with and 150 without postoperative recurrence.

The enrollment period extended from January 2022 to May 2024. Eligible participants were older than 18 years and fulfilled the diagnostic criteria for CRSwNP recommended by the European Position Paper on Rhinosinusitis and Nasal Polyps 2020 (1).

Because the study quantified both inflammatory and coagulation biomarkers as candidate predictors, exclusion criteria were designed to remove conditions and treatments that could confound these measurements: (1) incomplete clinical data; (2) fungal rhinosinusitis, allergic fungal rhinosinusitis, or nasal tumors; (3) systemic corticosteroid, antibiotic, or immunomodulatory treatment within one month before surgery; (4) acute infection; (5) age ≤18 years; (6) autoimmune disease or severe systemic comorbidity potentially affecting the inflammatory or coagulation biomarkers under investigation; and (7) a severe psychiatric or cognitive condition precluding reliable consent, clinical assessment, or longitudinal follow-up. Source-record review further confirmed that no patient in the final analysis cohort was receiving anticoagulant, antiplatelet, or nonsteroidal anti-inflammatory drug therapy before the preoperative blood draw. Postoperative treatment and endoscopic surveillance followed the standardized institutional protocol described below.

All participants were followed for a minimum of 2 years post-surgery. Recurrence of CRSwNP was defined as persistent clinical symptoms along with endoscopic and/or CT abnormalities, despite antibiotic treatment and oral corticosteroids, lasting at least two months (19). Based on the 2-year follow-up data, participants were divided into recurrence and nonrecurrence groups.

### 2.2 Data collection and processing

Demographic and clinical characteristics, laboratory parameters, and imaging and endoscopic scores were extracted from the Hospital Information System. WBC, PLT, and FIB were measured from a single blood-draw episode obtained after admission and within 24 h before surgery, and the systemic coagulation–inflammation index (SCI) was computed as FIB × PLT / WBC. Source-record review confirmed that no included patient had received anticoagulant, antiplatelet, or nonsteroidal anti-inflammatory therapy before this blood draw. Hypertension and diabetes mellitus were additionally abstracted as potential confounders of the coagulation–inflammation axis; data were available for 243 of 245 adults (99.2%), with both missing records in the training set. These comorbidities were incorporated into sensitivity analyses but not into the primary feature-selection process. Standardized tissue histopathology, tissue eosinophil counts, and serum albumin were not captured in this cohort.

### 2.3 Model construction and comparison

The original 270-case complete-case dataset had been partitioned by outcome-stratified random sampling at a 7:3 ratio using random seed 42. After the age-eligibility discrepancy was identified, the original assignments were retained and age-ineligible patients were removed within each subset rather than generating a new split. This yielded an adult training set of 170 patients and a same-center internal held-out set of 75 patients. Feature screening, model fitting, hyperparameter tuning, cross-validation, and threshold selection were performed exclusively in the training set.

#### 2.3.1 Baseline comparison by recurrence status and split-balance assessment

The main baseline table compares the recurrence and nonrecurrence groups. Continuous variables are reported as mean ± standard deviation and were compared using the Mann–Whitney U test, as several variables were non-normally distributed and a non-parametric approach was adopted throughout to avoid distributional assumptions. Categorical variables are reported as n/N (percentage) and were compared using the chi-square or Fisher exact test, as appropriate; variable-specific denominators are shown for variables with missing data. The training-versus-held-out balance table is provided as Supplementary Table S2.

#### 2.3.2 Construction of conventional prediction model

Univariable logistic regression was performed in the training set, and variables with *p* < 0.05 were considered for the multivariable model. Centered variance inflation factors were calculated from a design matrix containing an intercept. Because SCI is deterministically calculated from WBC, PLT, and FIB, it was excluded from the primary component model before VIF-based selection; any remaining predictor with VIF > 10 would then be removed sequentially. Three SCI sensitivity models were examined: a component model containing WBC, PLT, and FIB; a composite model containing SCI; and an expanded model containing WBC, PLT, FIB, and SCI. TT and PLR were included as a common adjustment set in all three models. Because the component and composite models were not nested within one another, likelihood-ratio tests were restricted to comparisons of each reduced model with the expanded model. Models were additionally summarized using AIC, five-fold cross-validated training AUC, held-out AUC, and Brier score. The final nomogram was refitted by conventional maximum-likelihood logistic regression using predictors that remained independently associated with recurrence in the multivariable component model. Its calibration slope and intercept were estimated in the internal held-out set by unpenalized logistic recalibration of the observed outcome on the logit of the predicted probability. For the comorbidity sensitivity analysis, hypertension and diabetes were forced simultaneously into a model containing WBC, PLT, and FIB. Because no recurrence occurred among diabetic patients in the comorbidity-complete training subset, Firth bias-reduced logistic regression was used in the 168 training patients with complete comorbidity data and evaluated in all 75 held-out patients. The adjusted model was compared with a Firth refit of the three-predictor model using the same 168 training patients, and their held-out AUCs were compared by the paired DeLong method.

#### 2.3.3 Construction and optimization of machine learning models

Six features identified by univariable screening—WBC, PLT, FIB, TT, SCI, and PLR—were supplied to ten classification algorithms: class-weighted L2-regularized logistic regression, Random Forest, Extra Trees, XGBoost, histogram gradient boosting, AdaBoost, radial-basis-function support vector machine, linear support vector machine, multilayer perceptron with early stopping, and k-nearest neighbors. The six-feature regularized logistic regression algorithm, referred to below as ML Logistic, was distinct from the conventional three-variable nomogram. Although SCI was excluded from the conventional multivariable model because of its deterministic relation to WBC, PLT, and FIB, it was retained in the exploratory machine-learning comparison to evaluate the predictive behavior of this prespecified nonlinear composite. Its inclusion was not interpreted as evidence that SCI contained biological information independent of its components. Algorithms requiring scaling used a StandardScaler within a pipeline. Hyperparameters were optimized in the training set by grid search with five-fold stratified cross-validation using ROC-AUC as the optimization metric.

#### 2.3.4 Model performance evaluation

Final performance was evaluated in the same-center internal held-out set using ROC-AUC with bootstrap 95% confidence intervals, AUPRC, Brier score, calibration slope and intercept, and decision-curve analysis. Calibration slope and intercept were estimated by unpenalized logistic recalibration of the observed outcome on the logit of each model’s predicted probability. Pairwise AUCs were compared using the paired DeLong method. Operating thresholds were selected by the Youden index in the training set and then applied unchanged to the held-out set. For model interpretation, ML Logistic was prespecified as the regularized analogue of the conventional model without consulting held-out performance. Its six-feature permutation importance was calculated in the training set as the mean decrease in ROC-AUC over 12 repeats. Because permuting SCI as a single feature breaks its deterministic relation with WBC, PLT, and FIB, this result was treated as an exploratory importance analysis only.

All analyses were performed using Python 3 and the software libraries listed in Supplementary Table S11. A two-sided *p* < 0.05 was considered statistically significant. Wald-type confidence intervals and *p* values are reported for the Firth sensitivity model. These analyses were exploratory; no multiplicity adjustment was applied.

## 3 Results

### 3.1 Study population and baseline characteristics

**Figure 1:**
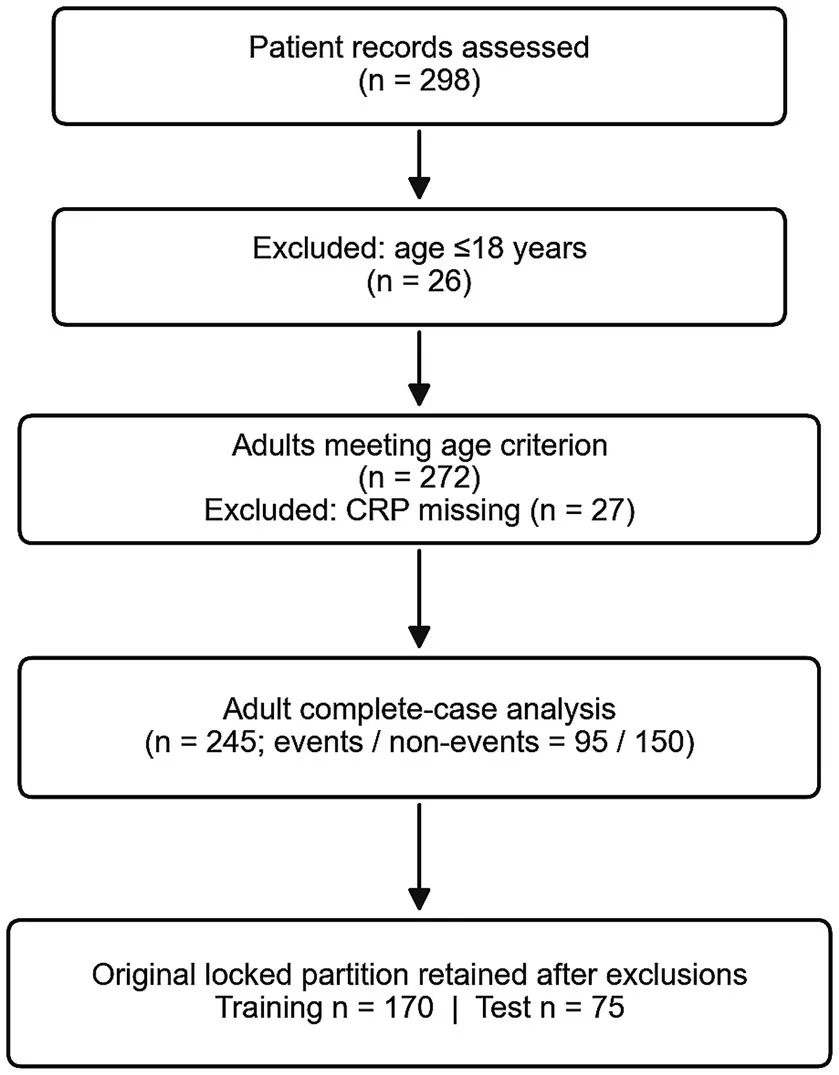
Study flowchart. Flowchart showing 301 physical worksheet rows, including 3 completely blank rows; 298 patient records assessed; exclusion of 26 patients aged ≤18 years and 27 age-eligible records with CRP unavailable for numeric complete-case analysis; the final 245-adult cohort; and retention of the original locked assignments (training *n* = 170; internal held-out *n* = 75).

The source worksheet contained 301 physical rows, of which 3 were completely blank, leaving 298 patient records for assessment. After excluding 26 patients aged ≤18 years and 27 age-eligible adults without a numeric CRP value for complete-case analysis, 245 adults remained. Recurrence occurred in 95/245 patients (38.8%). Removal of age-ineligible records within the original locked subsets yielded a training set of 170 patients and a same-center internal held-out set of 75 patients (Figure 1). Compared with the nonrecurrence group, the recurrence group had lower Lund–Mackay score, Lund–Kennedy score, neutrophil count, WBC, and TT, and higher monocyte percentage, basophil percentage, PLT, FIB, PLR, and SCI (all *p* < 0.05). Blood eosinophil count, eosinophil percentage, asthma, hypertension, and diabetes did not differ significantly between the two groups. Hypertension was present in 20/149 nonrecurrence patients and 10/94 recurrence patients (*p* = 0.658), whereas diabetes was present in 9/149 and 1/94 patients, respectively (*p* = 0.094). Comorbidity data were unavailable for two adults (Table 1).

**Table 1:** Baseline characteristics of adult patients stratified by postoperative recurrence.

Characteristics	Nonrecurrence (*n* = 150)	Recurrence (*n* = 95)	*P*
Age, years	50.99 ± 14.32	52.69 ± 14.49	0.283
Male sex, No. (%)	88/150 (58.7%)	54/95 (56.8%)	0.881
Smoking, No. (%)	45/150 (30.0%)	31/95 (32.6%)	0.770
Alcohol use, No. (%)	42/150 (28.0%)	25/95 (26.3%)	0.888
Disease duration, months	39.31 ± 62.00	42.64 ± 81.00	0.194
Allergic rhinitis, No. (%)	63/150 (42.0%)	44/95 (46.3%)	0.595
Asthma, No. (%)	5/150 (3.3%)	3/95 (3.2%)	1.000
Hypertension, No. (%)	20/149 (13.4%)	10/94 (10.6%)	0.658
Diabetes mellitus, No. (%)	9/149 (6.0%)	1/94 (1.1%)	0.094
Lund–Mackay score	12.46 ± 6.77	10.39 ± 6.40	0.022
Lund–Kennedy score	7.23 ± 3.60	6.11 ± 3.23	0.025
Nasal nitric oxide, ppb	429.07 ± 332.10	381.62 ± 244.64	0.561
Fractional exhaled nitric oxide, ppb	24.99 ± 21.42	23.46 ± 18.70	0.578
Monocyte count, ×10^9^/L	0.35 ± 0.14	0.35 ± 0.14	0.598
Monocyte percentage, %	5.33 ± 1.46	5.94 ± 1.63	0.006
Eosinophil count, ×10^9^/L	0.20 ± 0.18	0.17 ± 0.16	0.269
Eosinophil percentage, %	3.19 ± 2.89	3.03 ± 3.07	0.685
Basophil count, ×10^9^/L	0.03 ± 0.02	0.03 ± 0.02	0.473
Basophil percentage, %	0.44 ± 0.28	0.51 ± 0.29	0.033
Neutrophil count, ×10^9^/L	4.65 ± 2.47	3.87 ± 1.68	0.033
Neutrophil percentage, %	65.05 ± 11.22	63.64 ± 9.70	0.764
Lymphocyte count, ×10^9^/L	1.67 ± 0.61	1.50 ± 0.41	0.100
Lymphocyte percentage, %	26.00 ± 8.89	26.88 ± 8.55	0.969
White blood cell count, ×10^9^/L	6.90 ± 2.57	5.92 ± 1.92	0.002
Red blood cell count, ×10^12^/L	4.73 ± 0.54	4.69 ± 0.49	0.523
Hemoglobin, g/L	144.55 ± 16.81	143.52 ± 14.23	0.439
Platelet count, ×10^9^/L	223.13 ± 56.46	255.93 ± 57.42	<0.001
C-reactive protein, mg/L	4.69 ± 10.07	8.25 ± 16.37	0.070
Prothrombin time, s	10.91 ± 1.36	10.81 ± 0.74	0.887
Fibrinogen, g/L	2.95 ± 0.80	3.81 ± 1.24	<0.001
Prothrombin activity, %	102.65 ± 11.26	103.08 ± 9.24	0.912
International normalized ratio	0.99 ± 0.13	0.98 ± 0.07	0.792
D-dimer, mg/L	0.32 ± 0.38	0.33 ± 0.30	0.174
Activated partial thromboplastin time, s	27.07 ± 2.65	27.15 ± 2.13	0.407
Thrombin time, s	18.03 ± 1.43	17.44 ± 1.31	<0.001
Neutrophil-to-lymphocyte ratio	3.30 ± 2.92	2.73 ± 1.29	0.941
Platelet-to-lymphocyte ratio	146.68 ± 55.13	182.08 ± 61.43	<0.001
Monocyte-to-lymphocyte ratio	0.23 ± 0.11	0.24 ± 0.12	0.334
Eosinophil-to-lymphocyte ratio	0.12 ± 0.10	0.12 ± 0.13	0.553
Systemic inflammation response index	1.20 ± 1.46	1.02 ± 0.86	0.523
Systemic immune–inflammation index	734.04 ± 675.87	706.82 ± 406.19	0.060
Systemic coagulation–inflammation index	100.59 ± 37.16	169.01 ± 54.36	<0.001

Continuous variables are mean ± SD and were compared using the Mann–Whitney U test; categorical variables are n/N (%) and were compared using the chi-square or Fisher exact test. Hypertension and diabetes data were available for 243/245 adults, and the available denominators are shown in those rows. SD, standard deviation; AR, allergic rhinitis; LM, Lund–Mackay; LK, Lund–Kennedy; nNO, nasal nitric oxide; FeNO, fractional exhaled nitric oxide; WBC, white blood cell count; PLT, platelet count; CRP, C-reactive protein; PT, prothrombin time; FIB, fibrinogen; INR, international normalized ratio; APTT, activated partial thromboplastin time; TT, thrombin time; SCI, systemic coagulation–inflammation index; NLR, neutrophil-to-lymphocyte ratio; PLR, platelet-to-lymphocyte ratio; MLR, monocyte-to-lymphocyte ratio; ELR, eosinophil-to-lymphocyte ratio; SIRI, systemic inflammation response index; SII, systemic immune–inflammation index.

### 3.2 Independent risk factors and nomogram development

**Figure 2:**
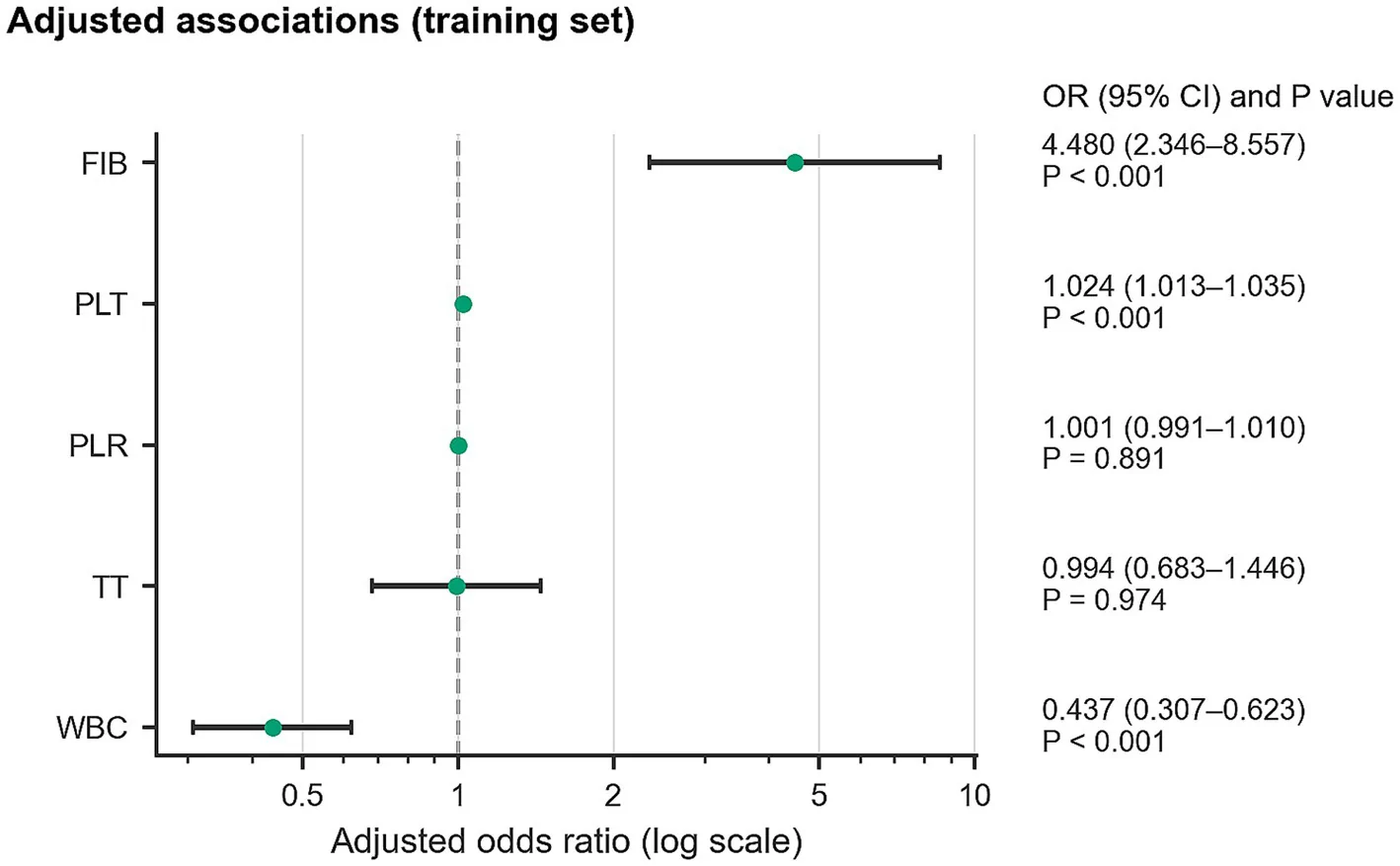
Multivariable associations with postoperative recurrence. Forest plot of adjusted odds ratios and 95% confidence intervals from the five-variable maximum-likelihood logistic model. WBC, white blood cell count; PLT, platelet count; FIB, fibrinogen; TT, thrombin time; PLR, platelet-to-lymphocyte ratio.

Univariable screening identified six variables meeting the prespecified *p* < 0.05 threshold: WBC, PLT, FIB, TT, SCI, and PLR. SCI was the only initial candidate exceeding the VIF threshold of 10 (VIF = 10.259); the corresponding VIFs were 5.209 for WBC, 3.625 for PLT, 6.060 for FIB, 1.406 for TT, and 1.448 for PLR. After SCI was excluded from the conventional component model, all remaining VIFs were ≤1.515 (Supplementary Table S3). In the resulting five-variable maximum-likelihood logistic model, WBC was inversely associated with recurrence (OR 0.437, 95% CI 0.307–0.623; *p* < 0.001), whereas PLT (OR 1.024, 95% CI 1.013–1.035; p < 0.001) and FIB (OR 4.480, 95% CI 2.346–8.557; *p* < 0.001) were positively associated. TT and PLR were not independently associated with recurrence (Table 2 and Figure 2).

**Table 2:** Univariable and multivariable logistic regression analyses of postoperative recurrence in the training set (*n* = 170).

	Univariable					Multivariable				
Variable	*β*	SE	Wald χ^2^	OR (95% CI)	*P*	*β*	SE	Wald χ^2^	OR (95% CI)	*P*
WBC	−0.169	0.076	4.909	0.844 (0.727–0.981)	0.027	−0.827	0.180	21.026	0.437 (0.307–0.623)	<0.001
PLT	0.011	0.003	14.289	1.011 (1.005–1.017)	<0.001	0.024	0.006	17.387	1.024 (1.013–1.035)	<0.001
FIB	1.023	0.210	23.816	2.781 (1.844–4.193)	<0.001	1.500	0.330	20.636	4.480 (2.346–8.557)	<0.001
TT	−0.374	0.127	8.656	0.688 (0.536–0.883)	0.003	−0.006	0.192	0.001	0.994 (0.683–1.446)	0.974
SCI	0.045	0.007	40.479	1.046 (1.032–1.061)	<0.001					
PLR	0.010	0.003	13.073	1.010 (1.005–1.016)	<0.001	0.001	0.005	0.019	1.001 (0.991–1.010)	0.891
Age	0.013	0.011	1.552	1.014 (0.992–1.035)	0.213					
Smoking	0.270	0.342	0.623	1.310 (0.670–2.558)	0.430					
Alcohol use	0.010	0.349	0.001	1.010 (0.510–2.000)	0.977					
Disease duration	0.000	0.003	0.008	1.000 (0.995–1.005)	0.927					
Allergic rhinitis	0.284	0.316	0.804	1.328 (0.714–2.468)	0.370					
Asthma	−0.980	1.129	0.753	0.375 (0.041–3.431)	0.385					
Lund–Mackay score	−0.041	0.024	2.869	0.960 (0.915–1.007)	0.090					
Lund–Kennedy score	−0.083	0.047	3.185	0.920 (0.840–1.008)	0.074					
nNO	−0.076	0.056	1.857	0.927 (0.830–1.034)	0.173					
FeNO	−0.005	0.009	0.317	0.995 (0.978–1.012)	0.573					
Monocyte count	−0.757	1.116	0.460	0.469 (0.053–4.183)	0.498					
Monocyte percentage	0.166	0.099	2.802	1.180 (0.972–1.432)	0.094					
Eosinophil count	−1.276	0.971	1.726	0.279 (0.042–1.873)	0.189					
Eosinophil percentage	−0.052	0.057	0.828	0.950 (0.850–1.061)	0.363					
Basophil count	0.050	0.093	0.292	1.051 (0.877–1.261)	0.589					
Basophil percentage	0.826	0.548	2.271	2.283 (0.780–6.683)	0.132					
Neutrophil count	−0.159	0.083	3.665	0.853 (0.725–1.004)	0.056					
Neutrophil percentage	−0.008	0.015	0.299	0.992 (0.963–1.021)	0.585					
Lymphocyte count	−0.439	0.306	2.063	0.645 (0.354–1.173)	0.151					
Lymphocyte percentage	0.011	0.018	0.366	1.011 (0.975–1.048)	0.545					
Red blood cell count	−0.215	0.309	0.485	0.806 (0.440–1.478)	0.486					
Hemoglobin	−0.001	0.010	0.012	0.999 (0.980–1.018)	0.914					
CRP	0.015	0.013	1.383	1.015 (0.990–1.041)	0.240					
PT	−0.186	0.176	1.117	0.830 (0.588–1.173)	0.291					
Prothrombin activity	0.016	0.016	1.102	1.016 (0.986–1.048)	0.294					
INR	−1.823	1.831	0.992	0.161 (0.004–5.840)	0.319					
D-dimer	0.484	0.450	1.152	1.622 (0.671–3.921)	0.283					
APTT	−0.043	0.066	0.434	0.957 (0.841–1.090)	0.510					
NLR	−0.107	0.077	1.941	0.899 (0.774–1.044)	0.164					
MLR	0.485	1.229	0.156	1.624 (0.146–18.056)	0.693					
ELR	−0.525	1.449	0.131	0.592 (0.035–10.133)	0.717					
SIRI	−0.144	0.146	0.977	0.866 (0.650–1.152)	0.323					
SII	−0.001	0.026	0.001	0.999 (0.950–1.051)	0.979					
Male sex	−0.224	0.317	0.499	0.800 (0.430–1.487)	0.480					

Odds ratios for nNO and SII are reported per 100-unit increase, and the odds ratio for basophil count is reported per 0.01 × 10^9^/L; all other continuous variables are reported per one-unit increase. Reference categories for male sex, smoking, alcohol use, allergic rhinitis, and asthma are female and no, respectively. Blank multivariable cells indicate variables that were not entered into the five-variable multivariable model. Units: age, years; disease duration, months; nNO and FeNO, ppb; leukocyte subtype counts, WBC, and PLT, ×10^9^/L; RBC, ×10^12^/L; hemoglobin, g/L; CRP and D-dimer, mg/L; PT, APTT, and TT, s; FIB, g/L; percentages, %. β, beta coefficient; SE, standard error; OR, odds ratio; CI, confidence interval; nNO, nasal nitric oxide; FeNO, fractional exhaled nitric oxide; WBC, white blood cell count; RBC, red blood cell count; PLT, platelet count; CRP, C-reactive protein; PT, prothrombin time; FIB, fibrinogen; INR, international normalized ratio; APTT, activated partial thromboplastin time; TT, thrombin time; SCI, systemic coagulation–inflammation index; NLR, neutrophil-to-lymphocyte ratio; PLR, platelet-to-lymphocyte ratio; MLR, monocyte-to-lymphocyte ratio; ELR, eosinophil-to-lymphocyte ratio; SIRI, systemic inflammation response index; SII, systemic immune–inflammation index.

SCI was excluded from the conventional multivariable model because it is deterministically calculated from WBC, PLT, and FIB and produced a VIF of 10.259. It was retained only in the exploratory six-feature machine-learning comparison to evaluate the predictive behavior of this prespecified nonlinear composite; this analysis was not interpreted as evidence that SCI contained information independent of its components.

**Figure 3:**
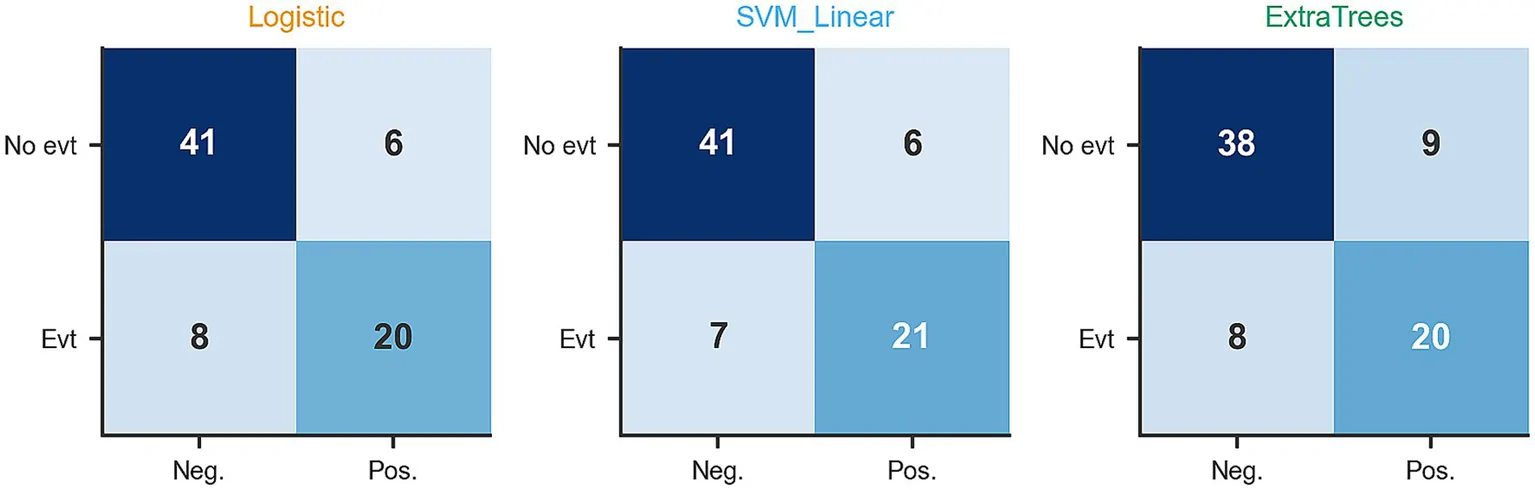
Nomogram for postoperative recurrence of CRSwNP. Nomogram based on *WBC*, *PLT*, and *FIB*. Points are summed to obtain predicted risk. The corresponding equation is logit(*p*) = −5.881 − 0.835 × *WBC* + 0.02397 × *PLT* + 1.516 × *FIB*.

The final conventional logistic nomogram included WBC, PLT, and FIB. Its equation was logit(p) = −5.881 − 0.835 × WBC + 0.02397 × PLT + 1.516 × FIB (Table 3 and Figure 3). The nomogram achieved a five-fold cross-validated AUC of 0.875 ± 0.021 in the training set and an AUC of 0.872 with a Brier score of 0.149 in the internal held-out set. The corresponding held-out calibration slope was 0.805 and the calibration intercept was −0.293 (Supplementary Table S6).

**Table 3:** Coefficients for the final three-predictor logistic nomogram fitted in the training set (*n* = 170).

Variable	*β*	SE	Wald χ^2^	OR (95% CI)	*P*
Intercept	−5.881	1.149	26.222	—	<0.001
WBC	−0.835	0.171	23.899	0.434 (0.310–0.606)	<0.001
PLT	0.024	0.005	22.868	1.024 (1.014–1.034)	<0.001
FIB	1.516	0.289	27.565	4.554 (2.586–8.020)	<0.001

Odds ratios are reported per one-unit increase. The intercept is reported on the log-odds scale; its transformed odds ratio is omitted because it has no clinical interpretation. WBC is measured in ×10^9^/L, PLT in ×10^9^/L, and FIB in g/L. β, beta coefficient; SE, standard error; OR, odds ratio; CI, confidence interval; WBC, white blood cell count; PLT, platelet count; FIB, fibrinogen.

Permutation importance was calculated for the separate six-feature, class-weighted, L2-regularized ML Logistic model, not for the nomogram. SCI had the largest apparent training ROC-AUC decrease (0.386), whereas the remaining single-feature decreases were close to zero. This pattern is expected when permuting a deterministic composite independently of its source variables and does not establish unique information (Supplementary Figure 1 and Supplementary Table S8).

In sensitivity analyses, the component, composite, and expanded models had held-out AUCs of 0.875, 0.879, and 0.872, respectively. Adding SCI to the component model improved training likelihood (likelihood-ratio χ^2^ = 5.537, 1 df; *p* = 0.0186) but did not improve held-out discrimination. Adding WBC, PLT, and FIB to the composite model did not improve training likelihood (χ^2^ = 0.227, 3 df; *p* = 0.973). Thus, SCI did not show stable incremental predictive value beyond its components across training fit, cross-validation, and held-out assessment (Supplementary Tables S4 and S5). In the separate comorbidity-complete Firth analysis (168 training patients), WBC (OR 0.441, 95% CI 0.315–0.616), PLT (OR 1.023, 95% CI 1.013–1.034), and FIB (OR 4.487, 95% CI 2.520–7.989) remained associated with recurrence (all *p* < 0.001), whereas hypertension (OR 1.258, 95% CI 0.333–4.755; *p* = 0.735) and diabetes (OR 0.027, 95% CI 0.0004–1.940; *p* = 0.098) were not. The wide diabetes interval reflects sparse data and separation. The adjusted model had a held-out AUC of 0.862 and Brier score of 0.151 versus 0.872 and 0.150 for the corresponding complete-case primary Firth model (paired DeLong *p* = 0.565; Supplementary Table S16).

### 3.3 Cross-validation and model selection in the training set

**Figure 4:**
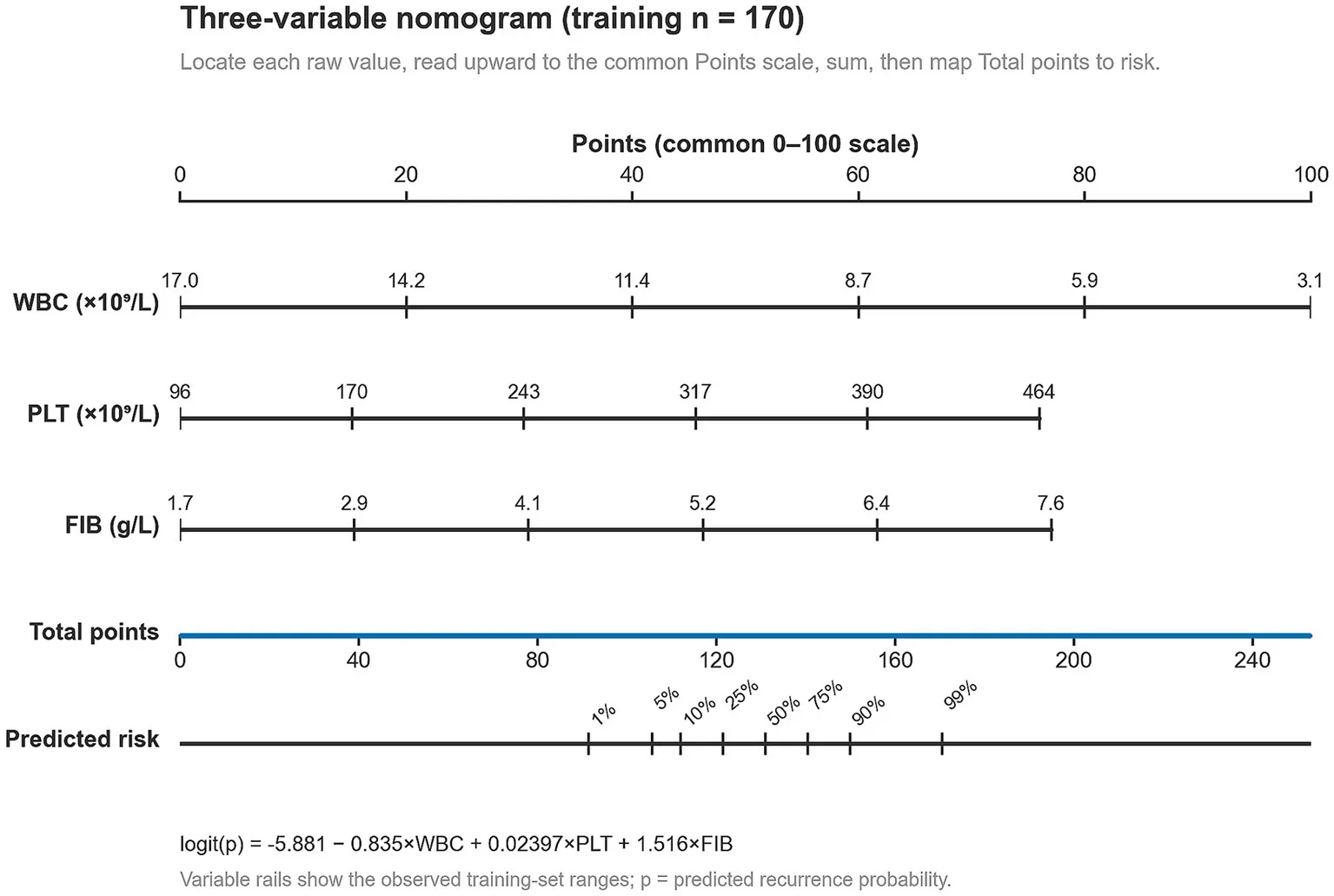
Model performance during training and in the internal held-out set **(A)** Five-fold stratified cross-validated AUC (mean ± SD) for all ten classification models in the adult training set (*n* = 170). **(B)** Precision–recall curves for the five models with the highest training CV-AUC in the internal held-out set. The horizontal dashed line indicates recurrence prevalence (28/75 = 0.373); all-model AUPRC values are reported in Table 4.

Ten machine-learning classifiers were evaluated by five-fold stratified cross-validation in the training set (Figure 4A). The highest CV-AUCs were obtained with ML Logistic (0.882 ± 0.028), linear SVM (0.882 ± 0.027), and Extra Trees (0.881 ± 0.035). ML Logistic denotes the six-feature, class-weighted, L2-regularized model and should not be confused with the conventional three-variable nomogram described in Section 3.2. The small differences in cross-validated AUC were not interpreted as evidence of algorithmic superiority. Full rankings and model settings are provided in Supplementary Tables S10, S11.

### 3.4 Precision–recall performance in the internal held-out set

Precision–recall curves for the five models selected by training CV-AUC—ML Logistic, linear SVM, Extra Trees, XGBoost, and radial-basis-function SVM—are shown in Figure 4B. Their held-out AUPRCs were 0.728, 0.734, 0.754, 0.740, and 0.731, respectively. Across all ten algorithms, Random Forest and HistGradientBoosting had the highest held-out AUPRCs, at 0.796 and 0.789, respectively. These held-out results were descriptive and were not used to reselect the models displayed in Figure 4B. Results for all algorithms are provided in Table 4 and Supplementary Table S12.

**Table 4:** Performance of ten classification models in the internal held-out set (*n* = 75).

Model	AUC (95% CI)	Sensitivity (95% CI)	Specificity (95% CI)	PPV (95% CI)	NPV (95% CI)	LR+ (95% CI)	LR− (95% CI)	Brier	Calibration slope	AUPRC
ML Logistic	0.871 (0.783–0.942)	0.714 (0.545–0.871)	0.872 (0.771–0.956)	0.769 (0.609–0.917)	0.837 (0.720–0.932)	5.595 (2.927–16.896)	0.328 (0.147–0.529)	0.155	0.643	0.728
Random Forest	0.856 (0.747–0.938)	0.750 (0.583–0.909)	0.872 (0.771–0.956)	0.778 (0.619–0.923)	0.854 (0.744–0.950)	5.875 (3.178–17.742)	0.287 (0.106–0.492)	0.139	0.997	0.796
XGBoost	0.810 (0.685–0.913)	0.714 (0.545–0.871)	0.872 (0.771–0.956)	0.769 (0.609–0.917)	0.837 (0.720–0.932)	5.595 (2.927–16.896)	0.328 (0.147–0.529)	0.156	0.557	0.740
SVM-RBF	0.867 (0.775–0.935)	0.750 (0.577–0.889)	0.872 (0.771–0.956)	0.778 (0.625–0.926)	0.854 (0.744–0.940)	5.875 (3.136–17.743)	0.287 (0.126–0.480)	0.151	0.862	0.731
SVM-Linear	0.870 (0.780–0.941)	0.750 (0.577–0.889)	0.872 (0.771–0.956)	0.778 (0.625–0.926)	0.854 (0.744–0.940)	5.875 (3.136–17.743)	0.287 (0.126–0.480)	0.152	0.579	0.734
MLP	0.855 (0.761–0.932)	0.607 (0.423–0.792)	0.851 (0.745–0.949)	0.708 (0.522–0.889)	0.784 (0.660–0.891)	4.077 (2.056–11.322)	0.462 (0.246–0.702)	0.173	1.938	0.714
HistGradientBoosting	0.842 (0.741–0.924)	0.750 (0.583–0.897)	0.872 (0.771–0.956)	0.778 (0.625–0.920)	0.854 (0.745–0.942)	5.875 (3.111–17.449)	0.287 (0.122–0.482)	0.155	0.699	0.789
AdaBoost	0.886 (0.807–0.950)	0.750 (0.583–0.909)	0.894 (0.800–0.976)	0.808 (0.652–0.947)	0.857 (0.745–0.952)	7.050 (3.642–28.596)	0.280 (0.106–0.477)	0.135	1.359	0.749
Extra Trees	0.881 (0.795–0.945)	0.714 (0.545–0.862)	0.809 (0.687–0.909)	0.690 (0.524–0.846)	0.826 (0.702–0.925)	3.730 (2.133–7.861)	0.353 (0.165–0.579)	0.166	2.006	0.754
KNN	0.821 (0.708–0.922)	0.036 (0.000–0.120)	1.000 (1.000–1.000)	1.000 (0.000–1.000)	0.635 (0.527–0.736)	Not estimable	0.964 (0.880–1.000)	0.153	0.914	0.721

All diagnostic metrics were calculated in the same-center internal held-out set (n = 75). Calibration slopes were estimated by unpenalized logistic recalibration. ML Logistic denotes the six-feature, class-weighted, L2-regularized machine-learning model and is distinct from the three-variable conventional nomogram. For KNN, LR+ is reported as not estimable because no false-positive classifications occurred at the training-derived threshold.

AUC, area under the receiver operating characteristic curve; CI, confidence interval; PPV, positive predictive value; NPV, negative predictive value; LR+, positive likelihood ratio; LR−, negative likelihood ratio; AUPRC, area under the precision–recall curve; ML, machine learning; SVM, support vector machine; RBF, radial basis function; MLP, multilayer perceptron; KNN, k-nearest neighbors.

### 3.5 Discrimination, calibration, and clinical utility in the internal held-out set

**Figure 5:**
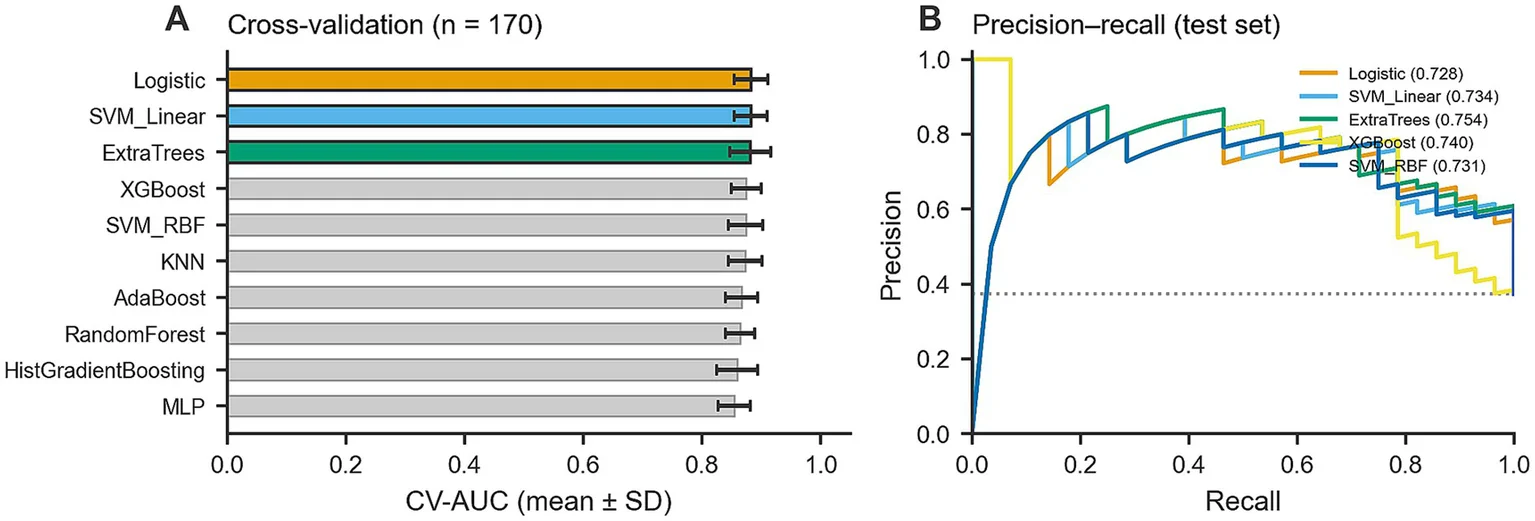
Discrimination, calibration, and decision-curve results in the internal held-out set. **(A)** ROC curves for the five models with the highest training CV-AUC. **(B)** Calibration plots for the same selected models; the 45° dashed line indicates perfect agreement. **(C)** Decision curves for the selected models, treat-all, and treat-none strategies. Curves are descriptive and require prospective validation.

Discrimination, calibration, and decision curves were evaluated in the same-center internal held-out set (Figure 5). Table 4 summarizes held-out classification metrics, and Supplementary Table S12 reports the full discrimination and calibration results.

#### 3.5.1 Discrimination

Among the ten machine-learning models, AdaBoost had the highest held-out AUC (0.886), followed by Extra Trees (0.881), ML Logistic (0.871), linear SVM (0.870), and radial-basis-function SVM (0.867). ML Logistic refers to the six-feature regularized machine-learning model rather than the conventional three-variable nomogram. These estimates were obtained from a single internal held-out set and were not interpreted as proof of algorithmic superiority.

#### 3.5.2 Calibration

Calibration results showed model-specific trade-offs rather than a uniformly superior algorithm. Using unpenalized logistic recalibration, AdaBoost had a Brier score of 0.135, a calibration slope of 1.359, and an intercept of −0.415. Random Forest had a Brier score of 0.139, a slope of 0.997, and an intercept of −0.156. ML Logistic had a Brier score of 0.155, a slope of 0.643, and an intercept of −0.667 (Figure 5B and Supplementary Table S12). These ML Logistic values apply to the six-feature regularized model. The conventional three-variable nomogram was evaluated separately and had a held-out calibration slope of 0.805 and intercept of −0.293. Calibration estimates were interpreted cautiously because the held-out set contained only 75 patients.

#### 3.5.3 Clinical utility

Decision-curve analysis showed that net benefit varied by model and threshold (Figure 5C). The curves are presented descriptively; they do not establish clinical effectiveness, and prospective validation of thresholds is required before clinical use.

No paired DeLong comparison with ML Logistic reached statistical significance (*p* = 0.166–0.923). Because no multiplicity correction or noninferiority margin was prespecified, nonsignificant differences were not interpreted as equivalence or noninferiority (Supplementary Tables S13, S14).

### 3.6 Classification performance at the Youden index threshold

**Figure 6:**
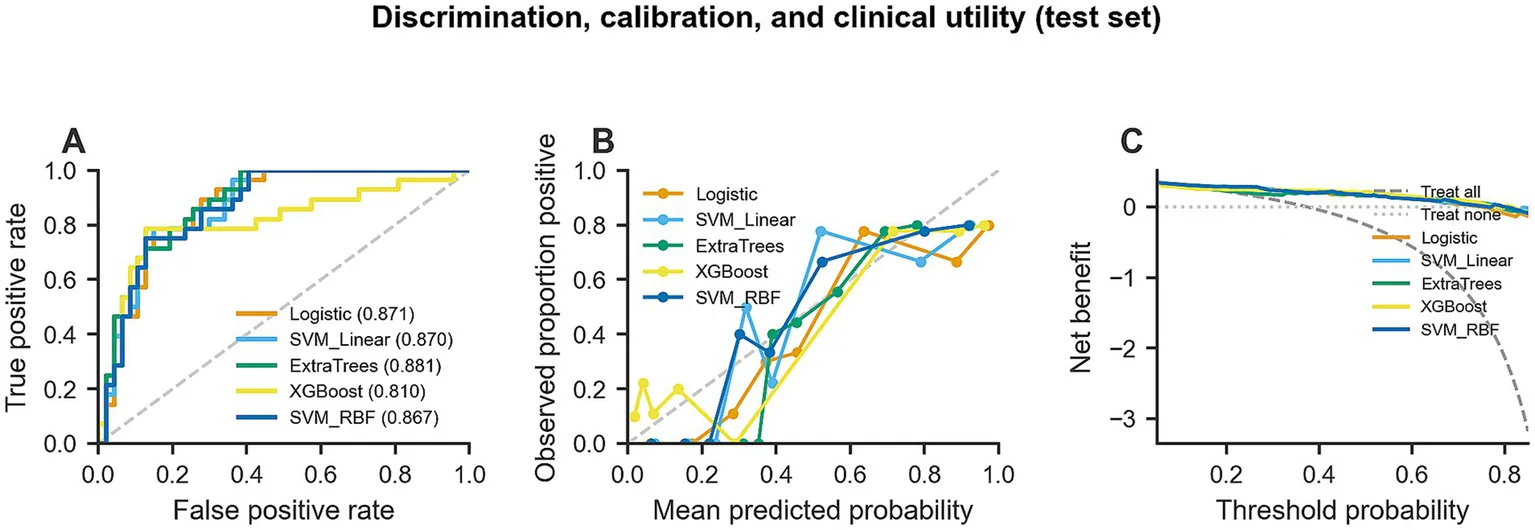
Confusion matrices for the three models with the highest training CV-AUC in the internal held-out set. Confusion matrices for ML Logistic, linear SVM, and Extra Trees using Youden-index thresholds derived in the training set. TN, true negative; FP, false positive; FN, false negative; TP, true positive.

Confusion matrices for the three models with the highest training CV-AUC are presented in Figure 6. Thresholds were selected using the Youden index in the training set and then applied unchanged to the internal held-out set. ML Logistic, linear SVM, and Extra Trees correctly classified 61/75, 62/75, and 58/75 patients, respectively; the corresponding counts are provided in Supplementary Table S15. At its fixed training-derived threshold, KNN detected only 1 of 28 recurrence cases, corresponding to a sensitivity of 0.036, while producing no false-positive classifications (specificity 1.000). This result indicates poor recurrence detection at that operating point; no alternative threshold was selected using the held-out data.

## 4 Discussion

CRSwNP is a heterogeneous disease characterized by chronic sinonasal inflammation, immune imbalance, epithelial-barrier dysfunction, and tissue remodeling (8, 20, 21). In addition to classic inflammatory pathways, abnormal activation of coagulation and impaired fibrinolysis can promote fibrin deposition. These interacting processes provide a rationale for evaluating routine inflammatory and coagulation markers together, but peripheral biomarkers cannot substitute for direct tissue characterization.

The sinonasal mucosa in CRSwNP contains neutrophils, eosinophils, mast cells, monocytes, and lymphocytes. Neutrophil extracellular traps and proteases can contribute to epithelial injury and remodeling (22–25). Eosinophils release cytotoxic granule proteins and type 2 cytokines that can amplify local inflammation (26–28). A distinct monocyte-derived macrophage population has also been associated with recurrent disease (29).

Blood and tissue eosinophilia are established but noninterchangeable markers in CRSwNP. Prior cohorts have linked preoperative blood eosinophils or tissue-eosinophilic phenotypes to recurrence, while other work has shown only weak correspondence between circulating and tissue eosinophils (30–32). In our adult cohort, neither Eos# nor Eos% differed by outcome (*p* = 0.269 and *p* = 0.685), and neither was significant in training-set univariable analysis. Forced-entry sensitivity models also showed no independent association (Eos# *p* = 0.566; Eos% *p* = 0.466) or improvement in held-out AUC. These negative findings may reflect endotypic heterogeneity, the small single-center sample, and the absence of tissue eosinophil measurements. The unusually low recorded asthma prevalence may additionally reflect same-center referral selection or incomplete retrospective documentation. The findings should not be interpreted as evidence against eosinophilic biology in CRSwNP.

Platelets participate in hemostasis, inflammation, and immune regulation (33), and prior studies have associated PLR with CRSwNP recurrence (34). In our data, PLR was associated with recurrence in univariable analysis but was not independently associated after platelet count and fibrinogen were included. Importantly, the lack of standardized polyp histopathology, tissue inflammatory-cell counts, and albumin prevented direct evaluation of tissue remodeling and local endotypes; peripheral indices should therefore be regarded as complementary, not substitutive, markers.

Among 245 adult complete cases, 95 (38.8%) experienced recurrence. After excluding the deterministic SCI composite from the primary component model, WBC was inversely associated with recurrence, whereas PLT and FIB were positively associated; TT and PLR were not independently associated.

FIB, a central coagulation marker, may reflect hypercoagulability and profibrotic activity, and coagulation-related mediators have been implicated in CRSwNP remodeling (35–37). The strong adjusted association of FIB supports further investigation, but the observational design does not establish a causal mechanism. In a sensitivity analysis, forcing hypertension and diabetes into a bias-reduced model did not materially alter the WBC, PLT, or FIB estimates or improve held-out discrimination. This supports numerical robustness to the two measured comorbidities, but residual confounding cannot be excluded because comorbidity data were unavailable for two patients and diabetes was rare. The lower Lund–Mackay and Lund–Kennedy values observed in recurrent patients were contrary to usual clinical expectations and were not stable independent predictors; this may reflect selection, measurement timing, or residual confounding and should not be interpreted as a protective effect.

SCI was associated with recurrence in univariable analysis and had the largest single-feature permutation score in the six-feature ML Logistic model. However, SCI equals FIB × PLT / WBC exactly. Its apparent importance therefore reflects the behavior of a nonlinear composite rather than independent biological information, and single-feature permutation can exaggerate that importance by breaking the deterministic identity.

SCI has been examined as a coagulation–inflammation marker in acute coronary syndrome, chronic obstructive pulmonary disease, infective endocarditis, and obstructive sleep apnea (17, 18, 38, 39). These applications support its plausibility as a summary measure but do not establish disease-specific mechanistic information in CRSwNP.

The SCI sensitivity analyses produced mixed results: adding SCI improved training likelihood but not held-out discrimination, while the compact composite model performed similarly to the component model. SCI may offer a parsimonious representation of its components, but the present data do not establish stable incremental predictive value. External validation and prespecified functional-form comparisons are required.

We also compared a conventional nomogram with ten machine-learning algorithms, an approach increasingly used for clinical risk prediction (40, 41). ML Logistic ranked highest by training CV-AUC, whereas AdaBoost had the highest held-out AUC and lowest Brier score and Random Forest had the highest AUPRC. These metric-specific trade-offs and the small single-center sample do not support a claim that any algorithm is uniformly superior.

The refitted three-variable nomogram is transparent and uses routine preoperative measurements obtained during the same blood-collection episode within 24 h before surgery. Its performance remains internally validated only, and it should be regarded as a research risk-stratification tool pending external validation and clinical-impact evaluation.

The present results may inform future hypothesis generation and prospective validation. They do not justify intensifying or simplifying treatment or follow-up on the basis of the model alone, because no decision threshold or model-guided intervention was prospectively evaluated.

Several limitations require emphasis. First, this was a retrospective single-center cohort with a single locked internal split and no external validation. Second, complete-case analysis excluded 27 age-eligible records because CRP was unavailable for numeric analysis and may introduce selection bias. Third, standardized tissue histopathology, tissue eosinophil counts, and albumin were unavailable, limiting endotype and remodeling assessment. Fourth, hypertension and diabetes were abstracted retrospectively for 243/245 adults; two training records remained unavailable, and diabetes was present in only 10 patients, including one recurrence. The resulting separation required Firth estimation and left wide confidence intervals, so cardiovascular and metabolic residual confounding cannot be excluded. Fifth, asthma was recorded in only 8/245 adults, substantially limiting power to assess this established risk factor. Sixth, SCI is structurally derived from FIB, PLT, and WBC, complicating coefficient and permutation-based interpretation. Finally, model comparisons were exploratory, with limited hyperparameter searches and no multiplicity adjustment or noninferiority design.

Future studies should use multicenter prospective cohorts, standardized tissue and blood phenotyping, complete prespecified cardiovascular and metabolic comorbidity capture, repeated or nested resampling, and independent external validation. These studies should also evaluate whether blood eosinophils, tissue eosinophilia, and coagulation markers provide complementary prognostic information across CRSwNP endotypes.

## 5 Conclusion

In this single-center retrospective adult cohort, WBC, PLT, and FIB were independently associated with postoperative recurrence, and a three-variable nomogram showed promising internal discrimination. SCI should be interpreted as a deterministic nonlinear composite of these measurements; stable incremental predictive value was not established. Prospective external validation with tissue phenotyping and comprehensive comorbidity data is required before clinical application.

## Data Availability

The original contributions presented in the study are included in the article/Supplementary material, further inquiries can be directed to the corresponding author/s.
