# Supplementary material for: Development and internal validation of a nomogram and machine-learning models for postoperative recurrence in adult patients with chronic rhinosinusitis with nasal polyps
Source: Front Med (Lausanne). 2026 Aug 20;13:1884376. doi: 10.3389/fmed.2026.1884376 (PMC13538003; doi:10.3389/fmed.2026.1884376)
Supplement: Supplementary file 1 [file Supplementary_file_1.DOCX]

**Supplementary Material**

**Supplementary Methods and reproducibility**

The primary analysis included 245 adults aged >18 years. The original outcome-stratified assignments from the locked 270-case partition were retained, then age-ineligible records were removed within each subset, leaving 170 training and 75 internal held-out patients. All feature screening, tuning, cross-validation, threshold selection, and permutation analyses used the training set only.

Centered VIFs used a design matrix containing an intercept. SCI was structurally excluded from the primary component model because SCI=FIB×PLT/WBC. Three SCI sensitivity models were examined using TT and PLR as common adjustment variables. Because the component and composite models were not nested within one another, likelihood-ratio tests were restricted to comparisons of each reduced model with the expanded model. Blood eosinophil count, eosinophil percentage, and asthma were separately forced into the primary nomogram as sensitivity analyses. Pairwise AUC comparisons used the paired DeLong method.

Hypertension and diabetes data were retrospectively available for 243/245 adults. Both unavailable records belonged to the training set, leaving 168 comorbidity-complete training patients and all 75 held-out patients. Because no recurrence occurred among diabetic patients in the complete training subset, conventional maximum-likelihood estimation exhibited complete separation. Firth bias-reduced logistic regression was therefore used with WBC, PLT, FIB, hypertension, and diabetes entered simultaneously. The held-out AUC was compared with that of a corresponding three-predictor Firth model fitted to the same 168 training patients using the paired DeLong method.

The complete pipeline is executed from the analysis-project root with `python -m analysis.run_all`. Software versions, random seed, partition handling, and analysis settings are reported in Supplementary Table S11.

## **Supplementary Table S1. Study-flow counts**

| **Step** | **Description** | **n** |
| --- | --- | --- |
| 1 | Source worksheet rows | 301 |
| 2 | Completely blank worksheet rows excluded | 3 |
| 3 | Patient records assessed | 298 |
| 4 | Excluded (outcome missing) | 0 |
| 5 | Excluded (age ≤18 years) | 26 |
| 6 | Adults meeting the age criterion | 272 |
| 7 | Excluded (incomplete covariate data; CRP in current dataset) | 27 |
| 8 | Adult complete cases (analysis set) | 245 |
| 9 | Postoperative recurrence (events) | 95 |
| 10 | No postoperative recurrence (non-events) | 150 |
| 11 | Training set (original locked partition retained) | 170 |
| 12 | Internal held-out set (original locked partition retained) | 75 |

*Note. The source worksheet contained 301 physical rows, including 3 completely blank rows that were not patient records. The 27 CRP exclusions include blank and non-numeric left-censored entries under the existing complete-case rule.*

## **Supplementary Table S2. Baseline balance between adult training and held-out sets**

| **Variable** | **Training (n=170)** | **Internal held-out (n=75)** | **P** | **Statistical test** |
| --- | --- | --- | --- | --- |
| Age, years | 51.97 ± 14.85 | 50.92 ± 13.33 | 0.563 | Mann-Whitney U |
| Smoking, No. (%) | 50/170 (29.4%) | 26/75 (34.7%) | 0.503 | Chi-square |
| Alcohol use, No. (%) | 48/170 (28.2%) | 19/75 (25.3%) | 0.753 | Chi-square |
| Disease duration, months | 35.76 ± 61.56 | 51.57 ± 85.17 | 0.601 | Mann-Whitney U |
| Allergic rhinitis, No. (%) | 74/170 (43.5%) | 33/75 (44.0%) | 1 | Chi-square |
| Asthma, No. (%) | 5/170 (2.9%) | 3/75 (4.0%) | 0.703 | Fisher exact |
| Lund–Mackay score | 11.52 ± 6.65 | 11.96 ± 6.81 | 0.727 | Mann-Whitney U |
| Lund–Kennedy score | 6.68 ± 3.48 | 7.05 ± 3.56 | 0.45 | Mann-Whitney U |
| Nasal nitric oxide, ppb | 421.31 ± 318.61 | 386.55 ± 259.19 | 0.58 | Mann-Whitney U |
| Fractional exhaled nitric oxide, ppb | 23.71 ± 18.33 | 25.97 ± 24.48 | 0.935 | Mann-Whitney U |
| Monocyte count, ×10⁹/L | 0.35 ± 0.14 | 0.36 ± 0.12 | 0.41 | Mann-Whitney U |
| Monocyte percentage, % | 5.56 ± 1.64 | 5.57 ± 1.35 | 0.764 | Mann-Whitney U |
| Eosinophil count, ×10⁹/L | 0.19 ± 0.18 | 0.18 ± 0.16 | 0.963 | Mann-Whitney U |
| Eosinophil percentage, % | 3.12 ± 2.90 | 3.15 ± 3.09 | 0.77 | Mann-Whitney U |
| Basophil count, ×10⁹/L | 0.03 ± 0.02 | 0.03 ± 0.01 | 0.81 | Mann-Whitney U |
| Basophil percentage, % | 0.47 ± 0.29 | 0.46 ± 0.28 | 0.66 | Mann-Whitney U |
| Neutrophil count, ×10⁹/L | 4.37 ± 2.26 | 4.28 ± 2.15 | 0.586 | Mann-Whitney U |
| Neutrophil percentage, % | 65.05 ± 10.53 | 63.27 ± 10.92 | 0.251 | Mann-Whitney U |
| Lymphocyte count, ×10⁹/L | 1.57 ± 0.55 | 1.68 ± 0.51 | 0.106 | Mann-Whitney U |
| Lymphocyte percentage, % | 25.80 ± 8.59 | 27.56 ± 9.06 | 0.189 | Mann-Whitney U |
| White blood cell count, ×10⁹/L | 6.51 ± 2.45 | 6.53 ± 2.25 | 0.859 | Mann-Whitney U |
| Red blood cell count, ×10¹²/L | 4.69 ± 0.51 | 4.78 ± 0.52 | 0.162 | Mann-Whitney U |
| Hemoglobin, g/L | 143.45 ± 16.04 | 145.73 ± 15.37 | 0.344 | Mann-Whitney U |
| Platelet count, ×10⁹/L | 233.24 ± 62.04 | 241.75 ± 51.10 | 0.113 | Mann-Whitney U |
| C-reactive protein, mg/L | 6.14 ± 12.74 | 5.92 ± 13.56 | 0.658 | Mann-Whitney U |
| Prothrombin time, s | 10.89 ± 1.29 | 10.83 ± 0.80 | 0.715 | Mann-Whitney U |
| Fibrinogen, g/L | 3.30 ± 1.12 | 3.25 ± 0.99 | 0.859 | Mann-Whitney U |
| Prothrombin activity, % | 102.74 ± 10.72 | 102.99 ± 10.06 | 0.736 | Mann-Whitney U |
| International normalized ratio | 0.99 ± 0.12 | 0.98 ± 0.07 | 0.914 | Mann-Whitney U |
| D-dimer, mg/L | 0.34 ± 0.35 | 0.30 ± 0.35 | 0.093 | Mann-Whitney U |
| Activated partial thromboplastin time, s | 27.11 ± 2.42 | 27.07 ± 2.54 | 0.862 | Mann-Whitney U |
| Thrombin time, s | 17.71 ± 1.36 | 18.01 ± 1.50 | 0.216 | Mann-Whitney U |
| Systemic coagulation–inflammation index | 126.70 ± 58.01 | 128.08 ± 50.27 | 0.425 | Mann-Whitney U |
| Neutrophil-to-lymphocyte ratio | 3.20 ± 2.62 | 2.81 ± 1.95 | 0.208 | Mann-Whitney U |
| Platelet-to-lymphocyte ratio | 162.41 ± 62.42 | 155.87 ± 54.51 | 0.786 | Mann-Whitney U |
| Monocyte-to-lymphocyte ratio | 0.24 ± 0.13 | 0.22 ± 0.09 | 0.681 | Mann-Whitney U |
| Eosinophil-to-lymphocyte ratio | 0.12 ± 0.11 | 0.11 ± 0.12 | 0.438 | Mann-Whitney U |
| Systemic inflammation response index | 1.16 ± 1.39 | 1.06 ± 0.93 | 0.626 | Mann-Whitney U |
| Systemic immune–inflammation index | 741.02 ± 616.30 | 683.73 ± 510.09 | 0.561 | Mann-Whitney U |
| Male sex, No. (%) | 97/170 (57.1%) | 45/75 (60.0%) | 0.772 | Chi-square |

*Note. Continuous variables are mean ± SD; categorical variables are n/N (%). Tests are two-sided. Variable units match Table 1 in the main manuscript.*

## **Supplementary Table S3. Centered VIF diagnostics**

| **Stage** | **Variable** | **VIF** | **Excluded from primary model** | **Reason for exclusion** |
| --- | --- | --- | --- | --- |
| Initial univariable candidates (P < 0.05) | WBC | 5.2089 | False |  |
| Initial univariable candidates (P < 0.05) | PLT | 3.6248 | False |  |
| Initial univariable candidates (P < 0.05) | FIB | 6.0596 | False |  |
| Initial univariable candidates (P < 0.05) | TT | 1.4062 | False |  |
| Initial univariable candidates (P < 0.05) | SCI | 10.2588 | True | Deterministic composite of WBC, PLT, and FIB |
| Initial univariable candidates (P < 0.05) | PLR | 1.4478 | False |  |
| After excluding structural composite SCI | WBC | 1.2061 | False |  |
| After excluding structural composite SCI | PLT | 1.3329 | False |  |
| After excluding structural composite SCI | FIB | 1.5154 | False |  |
| After excluding structural composite SCI | TT | 1.4059 | False |  |
| After excluding structural composite SCI | PLR | 1.2394 | False |  |

*Note. VIFs include an intercept. SCI was the only initial candidate above 10; after structural exclusion of SCI, the maximum VIF was 1.515.*

## **Supplementary Table S4. SCI component, composite, and expanded sensitivity models**

| **Model** | **Variables** | **Predictors, n** | **AIC** | **BIC** | **CV AUC, mean** | **CV AUC, SD** | **Held-out AUC** | **Held-out Brier score** |
| --- | --- | --- | --- | --- | --- | --- | --- | --- |
| A Component | TT, PLR, WBC, PLT, FIB | 5 | 152.58 | 171.3948 | 0.8695 | 0.0211 | 0.8746 | 0.1486 |
| B Composite | TT, PLR, SCI | 3 | 143.2694 | 155.8126 | 0.8855 | 0.0395 | 0.8792 | 0.1474 |
| C Expanded | TT, PLR, WBC, PLT, FIB, SCI | 6 | 149.0427 | 170.9933 | 0.881 | 0.0332 | 0.8716 | 0.1496 |

*Note. AUC, area under the receiver operating characteristic curve; CV, cross-validation; AIC, Akaike information criterion; BIC, Bayesian information criterion.*

## **Supplementary Table S5. Nested likelihood-ratio tests**

| **Comparison** | **LR χ²** | **df** | **P** |
| --- | --- | --- | --- |
| C expanded vs A component: add SCI | 5.5372 | 1 | 0.0186 |
| C expanded vs B composite: add WBC, PLT, FIB | 0.2266 | 3 | 0.9732 |

*Note. LR, likelihood ratio; df, degrees of freedom. P values are two-sided.*

## **Supplementary Table S6. Nomogram-model internal performance**

| **Model** | **Variables** | **Predictors, n** | **AIC** | **CV AUC, mean** | **CV AUC, SD** | **Held-out AUC** | **Held-out Brier score** |
| --- | --- | --- | --- | --- | --- | --- | --- |
| Component sensitivity model | TT, PLR, WBC, PLT, FIB | 5 | 152.58 | 0.8695 | 0.0211 | 0.8746 | 0.1486 |
| Composite sensitivity model | TT, PLR, SCI | 3 | 143.2694 | 0.8855 | 0.0395 | 0.8792 | 0.1474 |
| Refitted adult primary nomogram | WBC, PLT, FIB | 3 | 148.6008 | 0.8753 | 0.0213 | 0.8723 | 0.1489 |

Note. AUC, area under the receiver operating characteristic curve; CV, cross-validation; AIC, Akaike information criterion. For the refitted adult primary nomogram, the internal held-out calibration slope was 0.805 and the calibration intercept was −0.293, estimated by unpenalized logistic recalibration.

## **Supplementary Table S7. Forced-entry blood eosinophil and asthma sensitivity analyses**

| **Model** | **Variables** | **Added predictor** | **Added β** | **Added OR** | **OR 95% CI, lower** | **OR 95% CI, upper** | **Added-variable P** | **LR χ² vs primary** | **LR-test P** | **Held-out AUC** | **Held-out Brier score** |
| --- | --- | --- | --- | --- | --- | --- | --- | --- | --- | --- | --- |
| Primary adult nomogram | WBC, PLT, FIB |  |  |  |  |  |  |  |  | 0.8723 | 0.1489 |
| Primary + Eos# | WBC, PLT, FIB, Eos# | Eos# | -0.7505 | 0.4721 | 0.0365 | 6.1146 | 0.5658 | 0.3383 | 0.5608 | 0.8701 | 0.1476 |
| Primary + Eos% | WBC, PLT, FIB, Eos% | Eos% | -0.052 | 0.9493 | 0.8254 | 1.0919 | 0.4664 | 0.5442 | 0.4607 | 0.8663 | 0.1482 |
| Primary + AS | WBC, PLT, FIB, AS | AS | -0.2609 | 0.7703 | 0.0494 | 12.0105 | 0.8523 | 0.0356 | 0.8503 | 0.867 | 0.1497 |

*Note. Eos# and Eos% were entered in separate models because they represent alternative expressions of the same cell lineage; neither was added to the primary nomogram.*

## **Supplementary Table S8. Exploratory permutation importance**

| **Variable** | **Mean AUC decrease** | **SD** |
| --- | --- | --- |
| SCI | 0.3856 | 0.0331 |
| FIB | 0.0001 | 0.0006 |
| TT | 0.0001 | 0.0008 |
| PLT | 0 | 0.0015 |
| WBC | -0 | 0.0004 |
| PLR | -0.0003 | 0.0012 |

*Note. Single-feature SCI permutation breaks its deterministic relation with WBC, PLT, and FIB and is not evidence of unique information.*

## **Supplementary Table S9. Machine-learning input features**

| **Feature** | **Univariable OR** | **Univariable P** | **Selection rule** |
| --- | --- | --- | --- |
| WBC | 0.8445 | 0.0267 | P < 0.05 |
| PLT | 1.0113 | 0.0002 | P < 0.05 |
| FIB | 2.7806 | 0 | P < 0.05 |
| TT | 0.6877 | 0.0033 | P < 0.05 |
| SCI | 1.0462 | 0 | P < 0.05 |
| PLR | 1.0104 | 0.0003 | P < 0.05 |

*Note. Features met the prespecified training-set univariable P<0.05 screening rule. OR, odds ratio.*

## **Supplementary Table S10. Model ranking by training cross-validated AUC**

| **CV-AUC rank** | **Model** | **CV AUC, mean** | **CV AUC, SD** | **Display group** |
| --- | --- | --- | --- | --- |
| 1 | ML Logistic | 0.8825 | 0.0284 | Top3_by_CV_AUC |
| 2 | Linear SVM | 0.8818 | 0.0274 | Top3_by_CV_AUC |
| 3 | Extra Trees | 0.8809 | 0.0348 | Top3_by_CV_AUC |
| 4 | XGBoost | 0.874 | 0.0254 | Top5_by_CV_AUC |
| 5 | RBF SVM | 0.8731 | 0.0285 | Top5_by_CV_AUC |
| 6 | KNN | 0.8719 | 0.0284 | Other |
| 7 | AdaBoost | 0.8661 | 0.0273 | Other |
| 8 | Random Forest | 0.8637 | 0.0243 | Other |
| 9 | HistGradientBoosting | 0.8588 | 0.0345 | Other |
| 10 | MLP | 0.8542 | 0.0269 | Other |

*Note. AUC, area under the receiver operating characteristic curve; CV, five-fold stratified cross-validation.*

## **Supplementary Table S11. Analysis configuration**

| **Item** | **Value** |
| --- | --- |
| Analysis timestamp | 2026-07-26T09:03:34 |
| Outcome | Postoperative recurrence (1 = recurrence) |
| Complete cases (n) | 245 |
| Training n | 170 |
| Internal held-out n | 75 |
| Original held-out fraction | 0.3 |
| Partition handling | Original 270-case stratified partition retained; age-ineligible records removed within subsets |
| Random state | 42 |
| Univariate alpha | 0.05 |
| ML feature count | 6 |
| Source worksheet rows | 301 |
| Completely blank rows excluded | 3 |
| Patient records assessed | 298 |
| Excluded age ≤18 years | 26 |
| Adults meeting age criterion | 272 |
| Top3 models (by CV-AUC) | ML Logistic, Linear SVM, Extra Trees |
| Top5 models (by CV-AUC) | ML Logistic, Linear SVM, Extra Trees, XGBoost, RBF SVM |
| Python | 3.14.3 |
| pandas | 2.3.3 |
| numpy | 2.4.3 |
| scipy | 1.17.1 |
| statsmodels | 0.14.6 |
| scikit-learn | 1.8.0 |
| xgboost | 3.2.0 |
| matplotlib | 3.10.8 |
| openpyxl | 3.1.5 |

*Note. Versions and settings correspond to the final adult-only analysis run.*

## **Supplementary Table S12. Held-out discrimination and calibration**

| **Model** | **Held-out Brier score** | **Held-out AUPRC** | **Calibration slope** | **Calibration intercept** |
| --- | --- | --- | --- | --- |
| ML Logistic | 0.1555 | 0.728 | 0.6434 | -0.6672 |
| Random Forest | 0.1391 | 0.7963 | 0.9970 | -0.1556 |
| XGBoost | 0.1559 | 0.7397 | 0.5571 | -0.2607 |
| RBF SVM | 0.1506 | 0.7305 | 0.8615 | -0.3328 |
| Linear SVM | 0.1516 | 0.7337 | 0.5785 | -0.4054 |
| MLP | 0.173 | 0.7137 | 1.9375 | 0.1644 |
| HistGradientBoosting | 0.1549 | 0.7886 | 0.6986 | -0.2586 |
| AdaBoost | 0.1348 | 0.7493 | 1.3593 | -0.4151 |
| Extra Trees | 0.1661 | 0.7535 | 2.0065 | -0.4655 |
| KNN | 0.1534 | 0.7206 | 0.9135 | -0.1655 |

Note. AUPRC, area under the precision–recall curve. Calibration slope and intercept were estimated by unpenalized logistic recalibration in the internal held-out set. Under this parameterization, slopes below 1 generally indicate overly extreme predictions, whereas slopes above 1 indicate predictions that are not sufficiently extreme. Estimates should be interpreted cautiously because the held-out set contained 75 patients.

## Supplementary Table S13. Paired DeLong comparisons versus ML Logistic

| **Reference model** | **Comparison model** | **DeLong P** |
| --- | --- | --- |
| ML Logistic | Random Forest | 0.6235 |
| ML Logistic | XGBoost | 0.1784 |
| ML Logistic | RBF SVM | 0.7154 |
| ML Logistic | Linear SVM | 0.923 |
| ML Logistic | MLP | 0.5568 |
| ML Logistic | HistGradientBoosting | 0.3725 |
| ML Logistic | AdaBoost | 0.4802 |
| ML Logistic | Extra Trees | 0.5158 |
| ML Logistic | KNN | 0.1658 |

*Note. Two-sided paired DeLong P values; no multiplicity adjustment.*

## **Supplementary Table S14. All pairwise DeLong comparisons**

| **Model 1** | **Model 2** | **P** |
| --- | --- | --- |
| ML Logistic | Random Forest | 0.6235 |
| ML Logistic | XGBoost | 0.1784 |
| ML Logistic | RBF SVM | 0.7154 |
| ML Logistic | Linear SVM | 0.923 |
| ML Logistic | MLP | 0.5568 |
| ML Logistic | HistGradientBoosting | 0.3725 |
| ML Logistic | AdaBoost | 0.4802 |
| ML Logistic | Extra Trees | 0.5158 |
| ML Logistic | KNN | 0.1658 |
| Random Forest | XGBoost | 0.3034 |
| Random Forest | RBF SVM | 0.7312 |
| Random Forest | Linear SVM | 0.6464 |
| Random Forest | MLP | 0.9874 |
| Random Forest | HistGradientBoosting | 0.6951 |
| Random Forest | AdaBoost | 0.2906 |
| Random Forest | Extra Trees | 0.4197 |
| Random Forest | KNN | 0.2146 |
| XGBoost | RBF SVM | 0.2453 |
| XGBoost | Linear SVM | 0.2051 |
| XGBoost | MLP | 0.4222 |
| XGBoost | HistGradientBoosting | 0.2549 |
| XGBoost | AdaBoost | 0.0657 |
| XGBoost | Extra Trees | 0.1281 |
| XGBoost | KNN | 0.8433 |
| RBF SVM | Linear SVM | 0.6493 |
| RBF SVM | MLP | 0.5958 |
| RBF SVM | HistGradientBoosting | 0.4827 |
| RBF SVM | AdaBoost | 0.4844 |
| RBF SVM | Extra Trees | 0.2583 |
| RBF SVM | KNN | 0.1577 |
| Linear SVM | MLP | 0.5635 |
| Linear SVM | HistGradientBoosting | 0.4092 |
| Linear SVM | AdaBoost | 0.508 |
| Linear SVM | Extra Trees | 0.4375 |
| Linear SVM | KNN | 0.1463 |
| MLP | HistGradientBoosting | 0.7726 |
| MLP | AdaBoost | 0.426 |
| MLP | Extra Trees | 0.2666 |
| MLP | KNN | 0.4004 |
| HistGradientBoosting | AdaBoost | 0.1249 |
| HistGradientBoosting | Extra Trees | 0.2506 |
| HistGradientBoosting | KNN | 0.6522 |
| AdaBoost | Extra Trees | 0.8307 |
| AdaBoost | KNN | 0.1021 |
| Extra Trees | KNN | 0.0468 |

*Note. Two-sided paired DeLong P values; no multiplicity adjustment.*

## **Supplementary Table S15. Confusion-matrix counts**

| **Model** | **Training Youden threshold** | **TN** | **FP** | **FN** | **TP** | **Held-out accuracy** |
| --- | --- | --- | --- | --- | --- | --- |
| ML Logistic | 0.5344 | 41 | 6 | 8 | 20 | 0.8133 |
| Linear SVM | 0.4263 | 41 | 6 | 7 | 21 | 0.8267 |
| Extra Trees | 0.4883 | 38 | 9 | 8 | 20 | 0.7733 |

*Note. Thresholds were selected by the Youden index in the training set and applied unchanged to the held-out set.*

## **Supplementary Table S16. Hypertension- and diabetes-adjusted sensitivity analysis**

**Panel A. Comorbidity prevalence by outcome**

| **Variable** | **Nonrecurrence** | **Recurrence** | **P** | **Available, n** | **Missing, n** |
| --- | --- | --- | --- | --- | --- |
| Hypertension | 20/149 (13.4%) | 10/94 (10.6%) | 0.658 | 243 | 2 |
| Diabetes mellitus | 9/149 (6.0%) | 1/94 (1.1%) | 0.094 | 243 | 2 |

**Panel B. Firth bias-reduced multivariable model in the complete training subset (n=168)**

| **Variable** | **OR (95% CI)** | **P** |
| --- | --- | --- |
| WBC | 0.441 (0.315–0.616) | <0.001 |
| PLT | 1.023 (1.013–1.034) | <0.001 |
| FIB | 4.487 (2.520–7.989) | <0.001 |
| Hypertension | 1.258 (0.333–4.755) | 0.735 |
| Diabetes mellitus | 0.027 (0.0004–1.940) | 0.098 |

**Panel C. Internal held-out performance**

| **Model** | **Training, n** | **Held-out, n** | **Held-out AUC** | **Held-out Brier score** | **DeLong P vs primary** |
| --- | --- | --- | --- | --- | --- |
| Primary Firth model | 168 | 75 | 0.8723 | 0.1499 |  |
| Primary + hypertension + diabetes | 168 | 75 | 0.8617 | 0.151 | 0.5646 |

*Note. Hypertension and diabetes status was available for 243/245 adults; the two unavailable records were both in the training set. Diabetes was sparse (10/243 overall, with one recurrence), and no diabetic recurrence occurred in the complete training subset. Firth estimation was used to address separation. CI, confidence interval; AUC, area under the receiver operating characteristic curve.*
